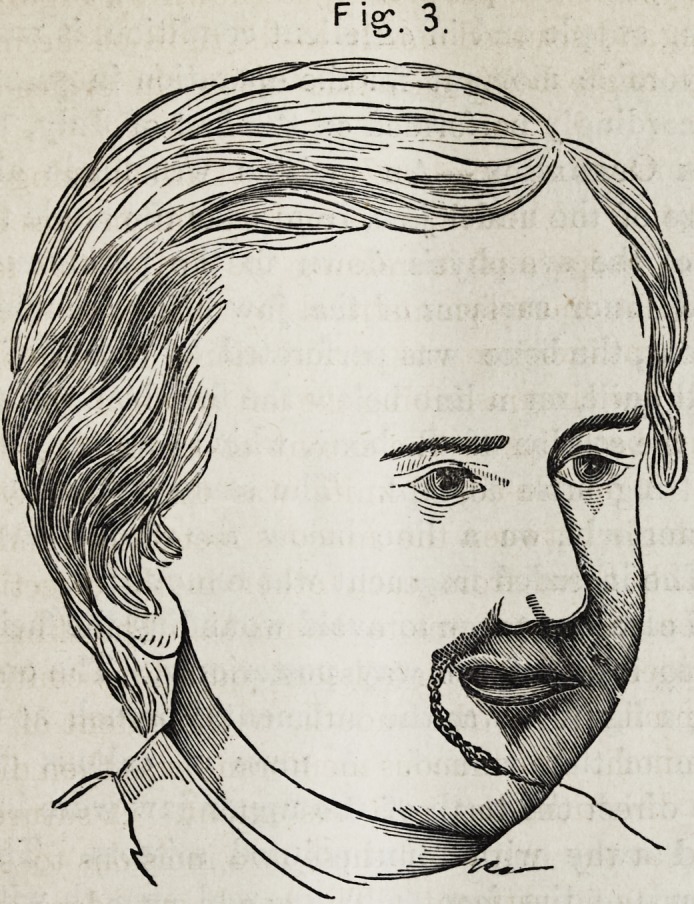# A Contribution to Reparative Surgery; Case of Destruction of the Right Half of Both Upper and Lower Lips, and Angle of the Mouth, with Closure of Jaws by Cicatricial Bands; Reconstruction of Mouth by a Succession of Plastic Operations, and Subsequent Relief of the Lower Jaw by Esmarck's Operation

**Published:** 1869-11

**Authors:** Gurdon Buck

**Affiliations:** Surgeon to New York Hospital and St. Luke's Hospital.


### SELECTED ARTICLES.

### ARTICLE V.

*A Contribution to Reparative Surgery ; Case of Destruction of the Right Half of both Upper and Lower Lips, and Angle of the Mouth, with Closure of Jaws by Cicatricial Bands ; Reconstruction of Mouth by a Succession of Plastic Operations, and Subsequent Relief of the Lower Jaw by Esmarck's Operation.*

By Gurdon Buck, M. D., Surgeon to New York Hospital and St. Luke's Hospital.

(With three wood-cuts.)

G. K. ., six years and four months old, of German parentage, resident of Williamsburg, Long Island, of fair complexion, and light hair, was admitted into St. Luke's Hospital in May, 1866.

The destruction of parts in this case appears, from the father's statement, to have been caused by cancrum oris occurring in the progress of typhoid fever, whether following the administration of mercury cannot be satisfactorily determined.

*Present Condition.*—One-half of the upper and two-fifths of the lower lip, with the angle of the mouth, on the right side, are gone, leaving the subjacent teeth and gum surface exposed. The adjacent cicatrized margin of the cheek is retracted and closely adherent to the upper and lower jaws, binding them together and preventing their separation from each other. The lining mucous membrane of the right cheek being destroyed, the free space between the teeth and cheek is obliterated. The remaining portions of the lips, on the left side, are shrunken in their vertical dimensions, leaving the teeth uncovered. The vermilion border of the upper lip terminates below the septum nasi, while that of the lower lip terminates below the right inferior canine tooth ; both lips are somewhat everted at their termination.

The columna nasi has been destroyed, leaving the inferior cicatrized margin of the septum exposed. Figure 1 shows the condition just described.

Fig. 1.

At the time of his admission a necrosed portion of the lower jaw, on the right side, was removed, and found to consist of the entire breadth of the jaw vertically, including three-fourths of an inch in length of its lower border, and containing in its upper border the entire alveolar socket of the second bicuspid tooth, with one-half the socket of the first bicuspid anteriorly and one-half the socket of first molar posteriorly.

Notwithstanding the loss of so considerable a portion of the jaw, the reproduction of the bone was so complete that subsequently no trace of deficiency could be detected by the finger passed along the lower margin of the jaw. Articulation is considerably affected, and the use of solid food rendered inadmissible by the closure of the teeth. His general health was pretty good, and steadily improved after admission into the hospital by the aid of generous diet and free out-door exposure.

First Operation.—*June* 20. After the administration of ether the right cheek was first detached, above and below, from the maxillæ, by applying the knife flatwise in contact with the surface of the bone, and continuing the dissection until all the resisting parts were liberated and the jaws could be separated from each other far enough to admit the thumb edgewise between the front teeth. The thin cicatrized edge of the skin, circumscribing the right angle of the mouth, was pared afresh preparatory to being adjusted to the lips. The next step of the operation was to prepare what remained of the lips and stretch them over to the right side, which was done as follows: Both lips were dissected up from the jaws, not only above and below, but also outward, on the left side, as far as the last molar teeth, after first dividing the mucous membrane on a line where it quits the gums to cover the cheeks. The upper lip was then detached by a horizontal incision, beginning below the septum nasi and carried through its entire thickness outward to an inch beyond the left angle of the mouth. The lower lip was also detached by a similar incision, parallel with the above, and crossing the upper part of the chin, and extending to the same distance. The free extremities of the bifurcated flap thus formed and which was lined with mucous membrane, were pared, and the whole flap stretched across to the right side, where the ends were adjusted to the edge of the cheek already prepared for the purpose. Pin sutures wound with yarn were inserted to secure the ends in place, and also fine interrupted sutures in close proximity to hold the edges of the horizontal wounds in accurate apposition. The parts thus transfixed, having been so extensively detached from their subjacent and neighboring connections, admitted of this new adjustment without any strain upon the sutures at any point.

Water dressings were directed to the face, and liquid nourishment ordered to be given through a tube. A moderate degree of inflammatory swelling followed the operation, but began to subside on the third day. On the fifth day nearly all of the sutures had been removed, union by first intention having taken place throughout most of the wound. Seventh day.—At the junction of the lip-flaps with the right cheek there is some ulceration; elsewhere union is complete. Appetite is good, and general condition satisfactory.

*July* 5. Parts have all healed. An attempt has been made to prevent the closure of the jaws by keeping a wooden wedge between the teeth during the process of cicatrization. It could, however, be borne only a part of the time, and ultimately accomplished nothing.

Fig. 2.

The newly constructed mouth, as shown by Figure, 2 is small in size, and situated mostly to the right of the median line, the left angle being on a line below the orifice of the left nostril.

Second Operation.—*September* 26. Patient being in excellent condition of health, a second operation was undertaken, for the purpose of increasing the size of the mouth, and rendering it more symmetrical by extending it at the left angle. An incision was made along the line of the vermilion border circumscribing the left angle of the mouth and involving both lips to the extent of about five-eighths of an inch. A double-edged knife was then inserted flatwise at the angle between the mucous membrane and skin, so as to detach them from each other in the direction in which the enlargement was to be made. The skin alone was first divided with strong scissors, on a line continuous with the commissure of the mouth, to the extent of three-fourths of an inch. The mucous membrane was then divided in the same direction, but to a less extent. A suture was then inserted at the angles of these two incisions to secure them in accurate adjustment. The newly-cut edges of skin and mucous membrane were next pared and matched to each other above and below, and brought into exact coaptation with fine interrupted sutures inserted close to each other. On the second day following the operation the alternate sutures were removed, and on the fourth day all the remaining sutures. The result of this operation was a more symmetrically-shaped mouth, though still too diminutive in size, its length scarcely exceeding one inch and a half.

Third Operation.—In the month of May, 1867, patient was readmitted into St. Luke's Hospital, and a third operation performed, in all respects similar to the second just described, for the purpose of lengthening the mouth still further in the direction of the left angle. The result was very satisfactory in rendering the mouth more symmetrical and improving the expression of the face. (See Fig. 3.)

Fig. 3.

Though the operations hitherto performed had effected all that was anticipated in repairing the disfigurement of the face and improving its appearance, our patient still suffered all the discomfort incident to the closure of the jaws. The only way of introducing soft food was by pushing it on with the finger between the teeth and cheek, on the left side, till it reached the mouth behind the last molar teeth. On the right side, at the angle of the mouth, and corresponding to the first bicuspid teeth, a tense cicatricial band commences and spreads over the entire cheek, obliterating its cavity and binding the jaws in close contact. to relieve this very serious difficulty, and facilitate the introduction of solid food into the mouth, it was proposed to resort to Esmarck's operation of establishing an artificial articulation anterior to the cicatricial band on the right side. Patient's general health being good, and the parts involved in the previous operations being supple and in excellent condition, it was considered a favorable moment for the operation in question and it was accordingly performed on the first of July, 1867.

Fourth Operation.—An incision was made along the lower edge of the under jaw, from near the angle to within an inch of the symphysis down to the periosteum. The outer and inner surfaces of the jaw being denuded to the same extent, the bone was perforated by a drill of the size of a small quill, on a line below the first bicuspid tooth, to facilitate the section of the bone, which was completed with strong cutting bone forceps. The same procedure was applied posteriorly, on a line below the second small molar tooth. The included fragment was removed.

Special care was taken to avoid wounding the facial artery by drawing it out of the way posteriorly. The only vessel requiring a ligature was the submental branch at the anterior angle of the wound. A mass of callous cicatricial tissue, in which the teeth of the upper jaw were imbedded, was pared away with blunt-pointed scissors. The newly divided ends of the jaw were gnawed smooth with Luer's rongeur forceps. The removed fragments measured one and a half inch in length. The anterior fragment of the jaw remaining in situ contained, in addition to the teeth belonging to the left half of the jaw, the incisors and canine of the right half, and could be separated from the upper jaw so as to admit a finger edgewise between the back teeth on the left side. This fragment also enjoyed the action of all the depressor muscles of the jaw which had not been disturbed at their insertions near the symphysis by the operation. A tent of lint of the thickness of the little finger was inserted with one end passing out at the right angle of the mouth, and the other through the wound below the jaw. The posterior half of the wound was closed with sutures. The hemorrhage during the operation was inconsiderable, and patient bore it well under the influence of ether. Water dressings were directed to be kept applied to the face and neck.

*July* 6.—Inflammatory swelling has been moderate, and is now on the decline. Patient is up and going about.

Nothing in the sequel of this case requires notice except the final result, which was as follows:—

The space left after the removal of a portion of the lower jaw has become obliterated by the approximation of the divided ends of bone, A limited motion of the left half of the jaw exists, and very much facilitates the introduction of food between the teeth. Patient, as well as his parents, appear much gratified with the improvement in his condition, especially the greater facility of feeding himself.—*American Jour. of the Med. Sciences.*

---

## Figures and Tables

**Fig. 1. f1:**
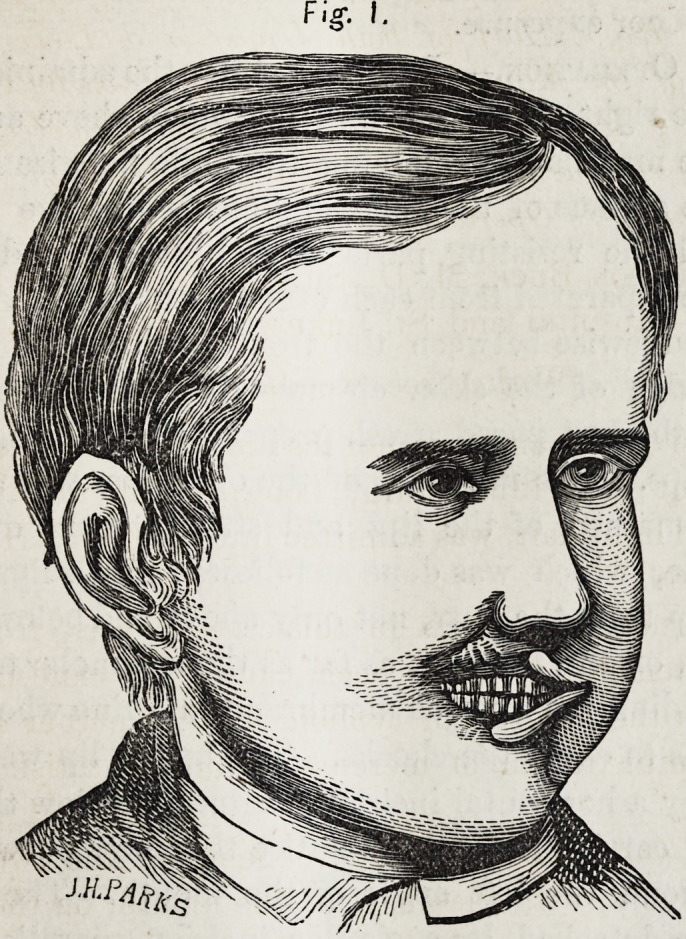


**Fig. 2. f2:**
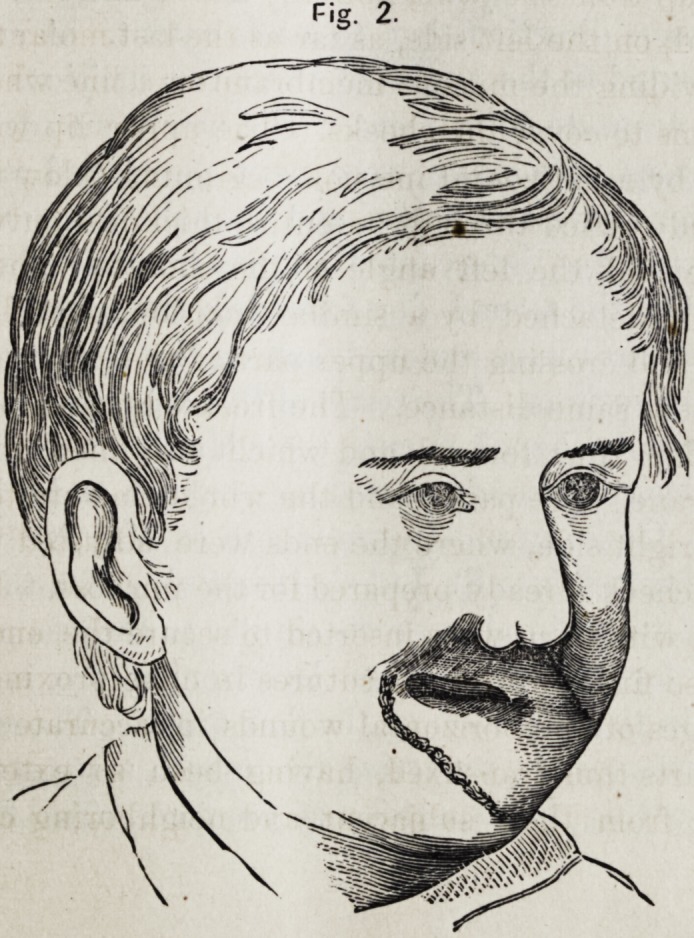


**Fig. 3. f3:**